# Creation of low cost, simple, and easy-to-use training kit for the dura mater suturing in endoscopic transnasal pituitary/skull base surgery

**DOI:** 10.1038/s41598-023-32311-2

**Published:** 2023-04-13

**Authors:** Yujiro Hattori, Eitaro Ishisaka, Shigeyuki Tahara, Koji Suzuki, Shinichiro Teramoto, Akio Morita

**Affiliations:** 1 Department of Neurological Surgery, Graduate School of Medicine, Nippon Medical School, 1-1-5 Sendagi, Bunkyo-ku, Tokyo, 113-8603 Japan; 2 Department of Anatomy and Neurobiology, Graduate School of Medicine, Nippon Medical School, Tokyo, Japan; 3 Department of Neurosurgery, Juntendo University School of Medicine, Tokyo, Japan

**Keywords:** Endocrine system and metabolic diseases, Neurological disorders

## Abstract

Training kits for laparoscopes for deep suturing under endoscopes are commercially available; however, previously reported training kits for endoscopic transnasal transsphenoidal pituitary/skull base surgery (eTSS) were not available in the market. Moreover, the previously reported low cost, self-made kit has the drawback of being unrealistic. This study aimed to create a low cost training kit for eTSS dura mater suturing that was as close to real as possible. Most necessary items were obtained from the 100-yen store ($1 store) or from everyday supplies. As an alternative to the endoscope, a stick-type camera was used. Through the assembly of the materials, a simple and easy-to-use training kit was created, which is almost identical to the actual dural suturing situation. In eTSS, a simple and easy-to-use training kit for dural suturing was successfully created at a low cost. This kit is expected to be used for deep suture operations and the development of surgical instruments for training.

## Introduction

Just fewer than 3000 cases of pituitary surgery are performed in Japan each year; among them, endoscopic transnasal transsphenoidal pituitary/skull base surgery (eTSS) is the most common, with the number of cases increasing every year^1^. In most cases, the surgeons make an incision through the pituitary dura and remove the tumor. If there is no arachnoid damage and no cerebrospinal fluid (CSF) leakage, it is empirically sufficient to apply fibrin glue after packing the fat in the excised cavity. However, in situations when intraoperative CSF leakage is observed, that method alone is not sufficient. Typical reconstruction methods include dural closure using various patch grafts including fascia patch grafts^2–7^, and covering the defect with a vascularized pedicled nasoseptal flap^8–11^. The former includes the gasket-seal method, but it cannot be used unless the bone margin around the dural defect remains. The AnastoClip has been reported as a simple method for dura mater suturing in eTSS^12^, but it has several limitations. The device is expensive and cannot be clipped properly without sufficient suture allowance. Therefore, classical suture techniques using a needle and thread are often necessary, but they are difficult due to the need for deep manipulation and require practice to master the technique.

Training kits for practicing laparoscopy with deep suturing under the endoscope can be constructed on our own at a low cost^13^, or they can be purchased commercially^14,15^. Meanwhile, no similar eTSS practice kits are available in the market. The creation of low cost training kit for suturing in eTSS was reported^16^, but it has the drawback of being unrealistic. Pituitary surgery simulators exist for purposes other than dural suturing training, but they are both large and expensive^17–21^, which is a barrier to skill acquisition. Therefore, this study aimed to create a dedicated dural suturing training kit for eTSS, which is as close to the real as possible, at the lowest possible cost.

## Materials and methods

The policy was designed to keep other costs as low as possible based on the premise that most surgeons would own electronic devices, such as PCs and monitors. Most of the necessary items were purchased at the 100-yen store ($1 store) or everyday items were used. Table 1 lists the items required to make the practice kit and their application.

**Table 1:** Materials and use application.

Materials	Intended use (recreation) for
Cutting board stand (Echo Kinzoku, Niigata, Japan)	Basic framework
Doorstop (Daiso Industries, Hiroshima, Japan)	Basic framework
50 cc syringe (Terumo, Tokyo, Japan)	Nasal corridor, and parafilm pasted
Adhesive agent (Cemedine, Tokyo, Japan)	Bond
Binder clip (Kokuyo, Osaka, Japan)	Fixation for syringe
Measuring spoon (Pearl Metal, Niigata, Japan)	Sphenoid sinus
White paint (Kusakabe, Saitama, Japan)	Sphenoid sinus coloring
Heavy paper (Workup, Kyoto, Japan)	Nasal septum
Fingertip (Kokuyo, Osaka, Japan)	Nasal cavity
Paper straw (Starbucks Coffee Japan, Tokyo, Japan)	Endoscope as an obstacle
Parafilm (Bemis, Chicago, IL, USA)	Dura mater
Nitrile glove (Kimberly-Clark, Neenah, WI, USA)	Free autologous graft
Rubber band (Kyowa, Osaka, Japan)	Parafilm fixation

The images were captured using a stick-type camera (approximately $40, BOEOC, Guangdong, China) and were connected to each individual’s electronic device for projection. The camera, which has a magnification range of 10× to 200×, an adjustable focal range from 10 to 500 mm, and a resolution of up to 1280 × 720 pixels, is also equipped with a built-in LED light that can be adjusted for brightness. The surgical instruments required were either the surgeons’ own needle-holder or inexpensive forceps for aquatic plant care (Gex, Osaka, Japan). Surgipro™ II (Covidien Japan, Tokyo, Japan) was used for suturing.

## Results

### Concept

**Figure 1:**
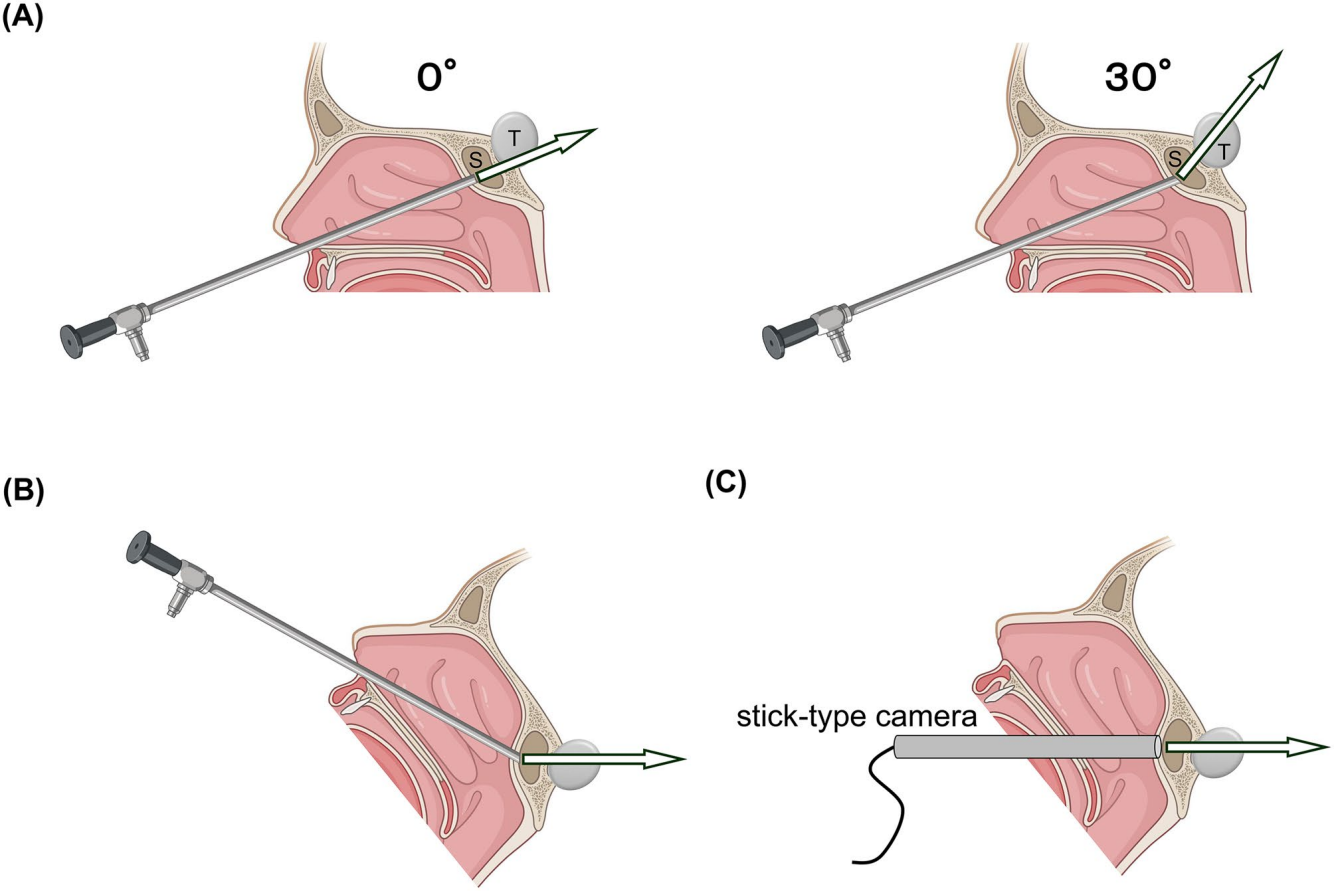
Schematic diagram of the midline sagittal section of the head showing the concept for making a training kit. (**A**) Conceptual diagram using an endoscope used in real surgery. The EndoArm endoscope system with a camera with a 0° field of view for manipulation in the nasal cavity and sphenoid sinus (S), and a camera with a 30° field of view for removal of tumor (T) inside the sella turcica were used. (**B**) Conceptual diagram in position in actual surgery. (**C**) Consideration of placement location of a stick-type camera for training kit. The illustrations were created with BioRender.com.

For eTSS, the EndoArm endoscope system (Olympus, Tokyo, Japan) is used with a camera with a 0° field of view for manipulation in the nasal cavity and sphenoid sinus at the Nippon Medical School Hospital. A camera with a 30° field of view is used for manipulation inside the sella turcica (Fig. 1A). Simultaneously, the endoscope is placed at the lower end of the nasal cavity to ensure the surgical instruments do not interfere with each other. The patient’s head position is elevated and adjusted so that the insertion angle of the device into the nasal cavity is about 30° from the horizontal plane (Fig. 1B). The 30° viewing angle projects almost horizontally, so that the goal can be achieved by positioning the stick-type camera as shown in Fig. 1C.

### Assembly

**Figure 2:**
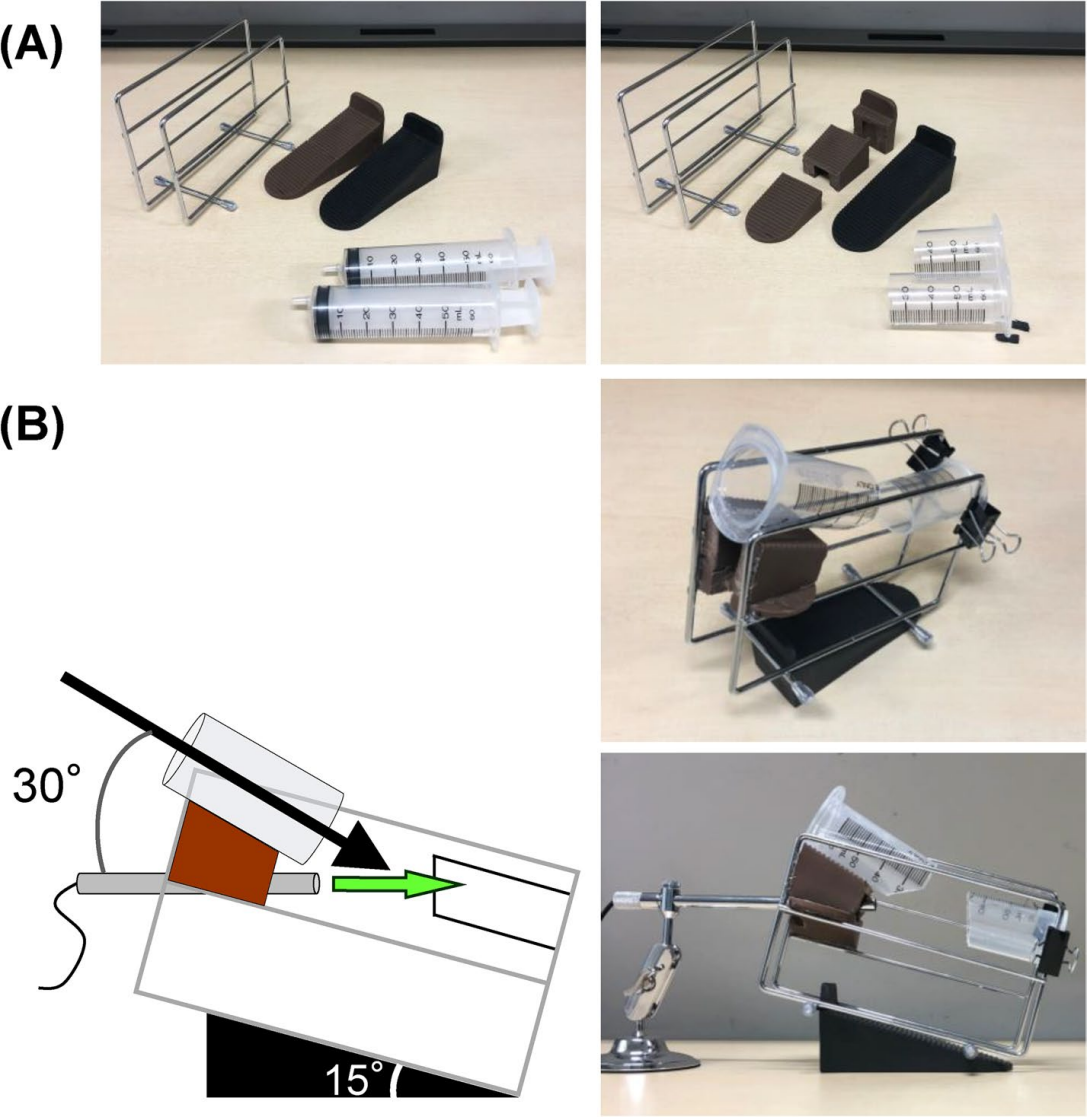
Materials of basic framework used for the training kit. (**A**) Materials for the basic framework. Left panel, before cutting; right panel, after cutting. (**B**) After assembly. Note that the initial concept was achieved.

**Figure 3:**
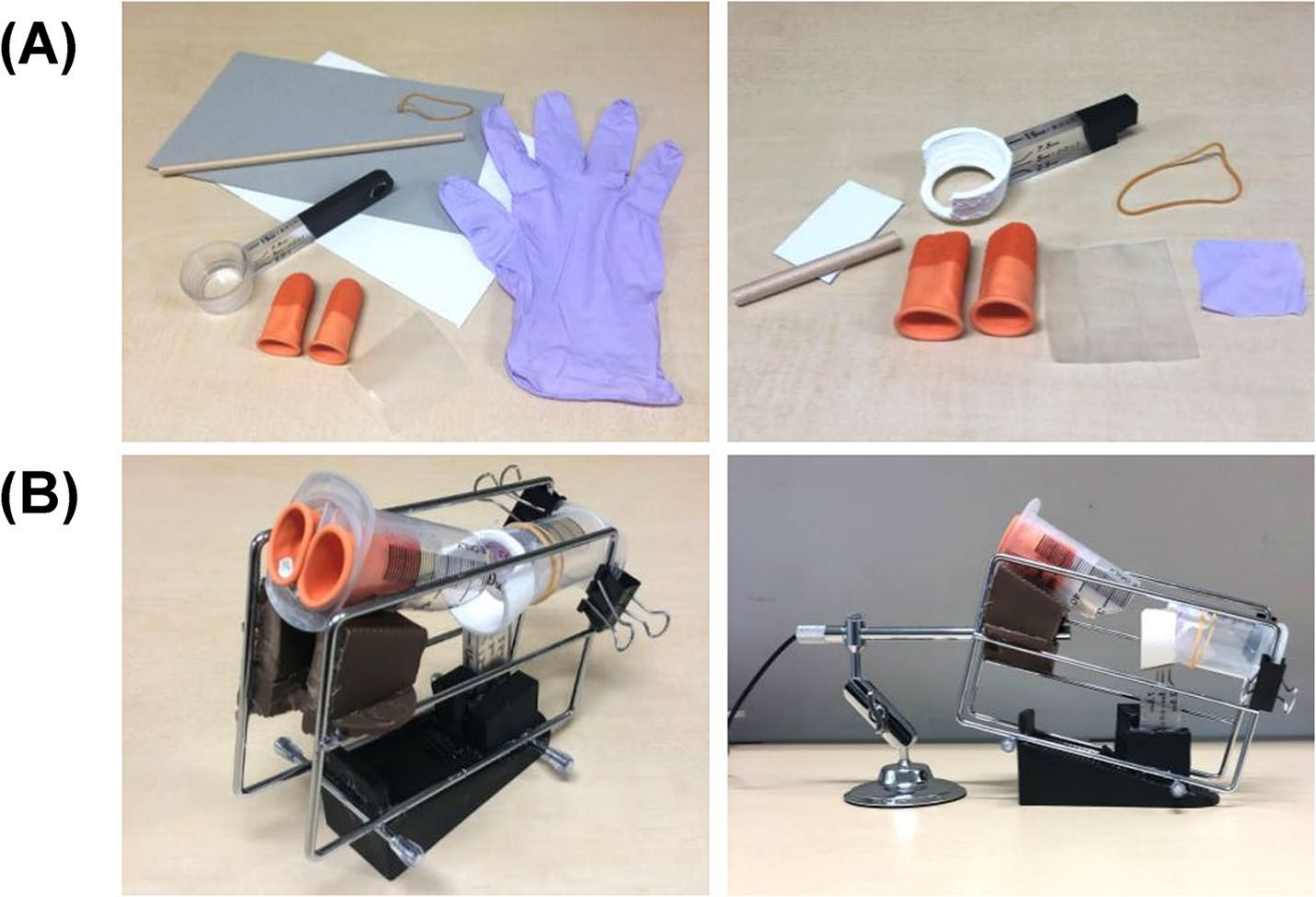
Other materials used for the training kit. (**A**) Materials for other objects. Left panel, before cutting; right panel, after cutting. The bottom of the measuring spoon was hollowed out, and the measuring part was colored white with paint to reproduce the sphenoid sinus. (**B**) After assembly.

To assemble the basic framework, the cutting board stand was prepared, and two doorstops (15° tilt) and two 50 cc syringes were cut (Fig. 2A). Following this, the items were assembled (Fig. 2B). The initial concept was achieved when the stick-type camera was installed. The remaining materials were prepared and cut as shown in Fig. 3A. The bottom of the measuring spoon was hollowed out, and the measuring part was painted white to reproduce the sphenoid sinus. These materials were then connected to the basic framework (Fig. 3B). The total cost was less than $10.

**Figure 4:**
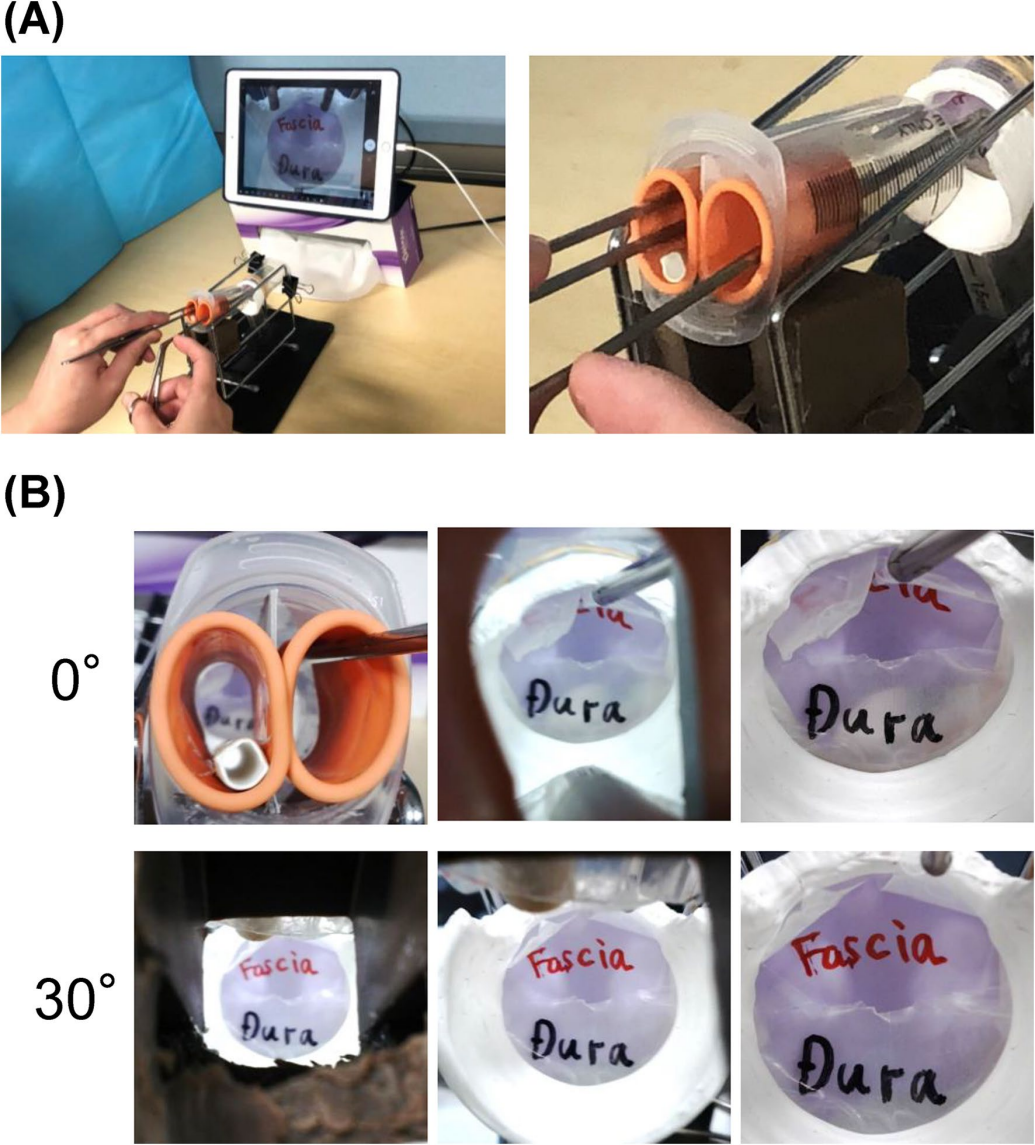
Training with equipment. (**A**) Left panel, Training with equipment; right panel, enlarged view at hand. (**B**) The image captured by the stick-type camera. Upper and lower panels, the 0° and 30° views, respectively.

Figure 4A shows the training procedure. Placing the paper straw in the position where the endoscope is normally located ensures that operability will be closer to that of actual surgery. The corridor of the instrument is narrower due to the syringe and the measuring spoon, which makes it closer to a genuine surgical situation. The image captured by the stick-type camera is shown in Fig. 4B. The 30° view looks up at the dura mater of the sella turcica from below, compared with the 0° view, which is when the endoscope is inserted through the nasal cavity.

### Training

**Figure 5:**
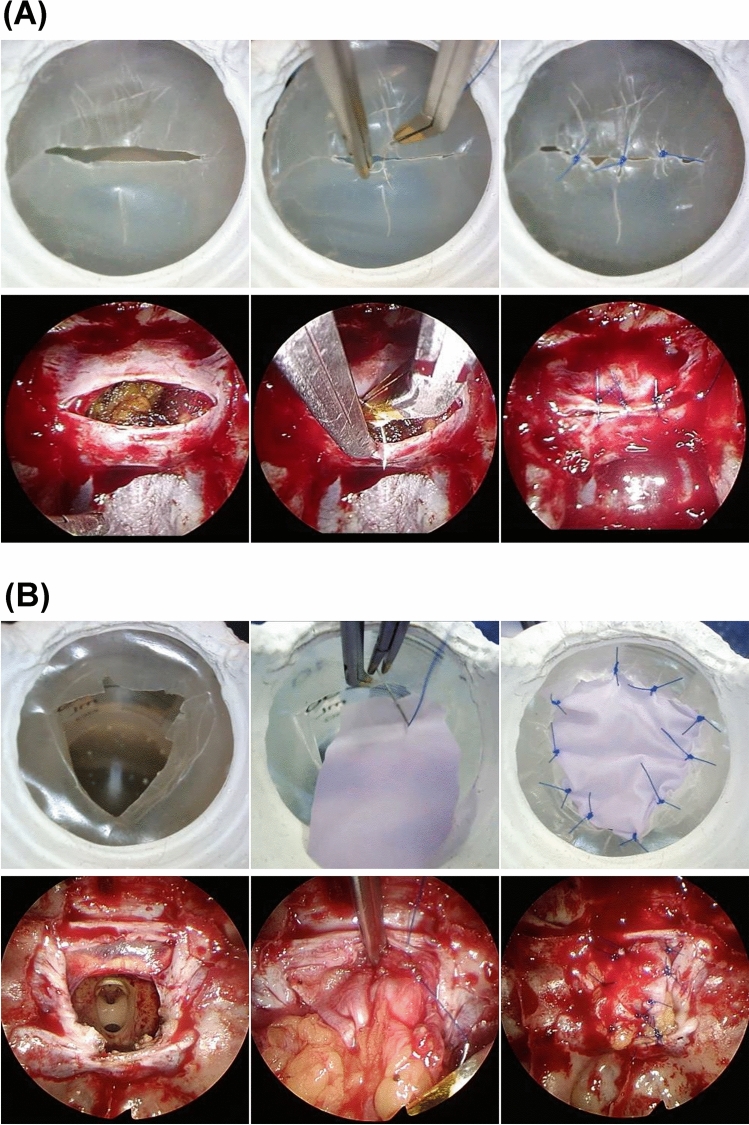
Actual training. Upper panels, the images of practicing; Lower panels, the surgical field envisioned. (**A**) Suture of the pituitary dura mater incised in a single horizontal letter. (**B**) Suture of a large incision of pituitary dura mater using a free graft.

The completed training kit was used to perform the suture practice. Like actual endoscopic surgery, the image displayed on the monitor is flat; hence, the experience is required for mastery but with practice, the surgeons become familiar with the device. Two typical practice scenes are shown in Fig. 5A,B.

Figure 5A shows a situation where the suture of the pituitary dura mater is incised in a single horizontal letter. Practicing this, for example, is useful for dural suturing in fenestration surgery for Rathke’s cleft cyst. The easy slip-knot technique was used, in which the suture is passed through the dura mater, ligated outside the body, and sent to the deep surgical field^22^.

Figure 5B shows an extended transsphenoidal surgery field with a wide incision in the pituitary dura. The dura is frequently shrunk in these conditions, and suturing the dura mater together is usually impossible; therefore, a free graft, such as the fascia lata, is placed on the underlay and sutured. This time, a nitrile glove cut into a square is used as a substitute for a free graft; hence, the sensation is slightly different from the actual fascia lata graft, but the technique is useful for suturing the graft and dura mater.

## Discussion

In this study, a low cost, simple, and easy-to-use training kit for dural suturing in endoscopic transnasal pituitary surgery was created. This total cost for practice was less than $50, including the cost of stick-type camera. Despite minor differences, such as the nasal cavity not extending similar to live patient, a situation was created that was almost identical to real surgery. Parafilm was used as the material for the dura mater, which was easily removable, and the feel of penetrating the needle was very close to the real dura mater. However, when tugged strongly with forceps, it has the disadvantage of stretching and deforming; hence, other good materials would be a better solution. The kit is useful for practicing dural sutures, but since the dural suture of the sella turcica often cannot be made completely watertight, auxiliary materials such as fat grafts and fibrin glue are needed to prevent spinal fluid leakage in real surgery.

When the pituitary dura is sutured, the endoscopic camera with a 30° field of view is placed at the lower end of the nasal cavity. Some pituitary surgeons use a 0° endoscope instead of a 30° endoscope. In these cases, trainees can remove the paper straw and insert a stick-type camera through the syringe that imitated the nasal cavity. In other words, this training kit can be used both in 0° and 30° fields of view.

**Figure 6:**
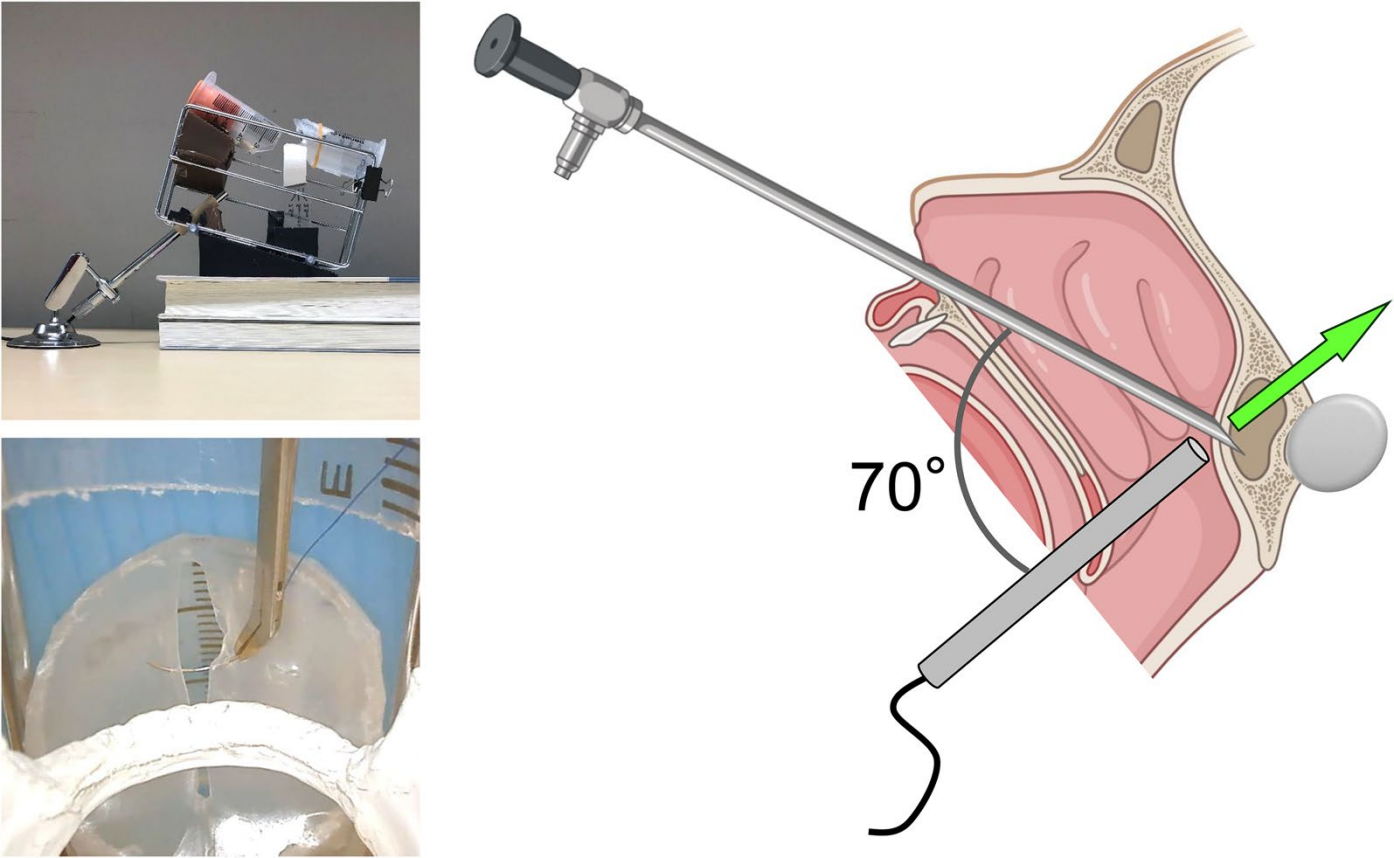
Observation of anterior skull base. This training kit can also simulate the vision of EndoArm with a camera with 70° field of view. The illustration was created with BioRender.com.

Although this training kit is low cost, simple, and easy-to-use, the angles are carefully calculated just like an actual surgery. EndoArm is mainly used with a camera with 0° and 30° field of view in eTSS; moreover, a camera with 70° field of view is occasionally used for observations toward the anterior skull base. As shown in Fig. 6, this training kit can also simulate the vision of EndoArm with a camera with 70° field of view. Therefore, the ability to observe at the same angle as an actual surgical endoscope suggests that this simple and easy-to-use training kit can not only be used for suturing exercises but also for developing good surgical instruments for suturing. In other words, although there are reports of the development of surgical equipment using expensive models^23,24^, there is a possibility that development can be done without using such high-class models.

Needle holders for the dura mater suturing in eTSS are very expensive, but if they are just used for practice, inexpensive forceps can be substituted if they have a firm grip on the forceps tip. Although low cost needle holders would be sufficient for training, the feel would be different, indicating that it is preferable to use the same ones in practice as in real surgery, if possible. Similarly, it is better to use the same sutures in training as in actual surgery. When suturing with the easy slip-knot method every time in training, the suture becomes shorter and shorter, which is a problem. If a large number of sutures were available, it would be preferable; however, although it is difficult to obtain, training can still be achieved with just one needle. The practice can be divided into a process for needle penetration and a process for ligation using the easy slip-knot method. For the ligation process alone, inexpensive threads, such as kite string, can be used for practice.

The training kit in this study was created by neurosurgeons who are already proficient in pituitary surgery. Therefore, there is a limitation that we could not evaluate the practice effect in inexperienced young surgeons. Measuring the time required for suturing before and after training may be preferred; however, it may not be appropriate because the suture time in real surgery is influenced by anatomical factors such as the size of the patient's nasal corridor and it is more important that the accurate sutures are reliable than fast procedures. However, it is expected that practicing with this kit will enable inexperienced young surgeons to become familiar with the deep suture operation. Despite inherent subjectivity, we experienced reduced stress while performing suturing in real surgery, and suturing time was shortened by practicing with this training kit (data not shown).

In conclusion, a simple and easy-to-use training kit for dural suturing in eTSS was successfully made at a little expense. We hope that this kit will be widely used in the future and that many pituitary surgeons will practice suturing, which will help raise the level of pituitary surgery. Additionally, it is expected that this kit can be used for developing surgical instruments without using high-class models.

## Data Availability

All data generated or analyzed during this study are included in this published article.

## References

[1] Hattori Y (2020). Pituitary surgery's epidemiology using a national inpatient database in Japan. Acta Neurochir. (Wien).

[2] Kitano M, Taneda M (2004). Subdural patch graft technique for watertight closure of large dural defects in extended transsphenoidal surgery. Neurosurgery.

[3] Leng LZ, Brown S, Anand VK, Schwartz TH (2008). "Gasket-seal" watertight closure in minimal-access endoscopic cranial base surgery. Neurosurgery.

[4] Ishii Y (2015). Fascia patchwork closure for endoscopic endonasal skull base surgery. Neurosurg. Rev..

[5] Garcia-Navarro V, Anand VK, Schwartz TH (2013). Gasket seal closure for extended endonasal endoscopic skull base surgery: Efficacy in a large case series. World Neurosurg..

[6] Tosaka M (2021). Single-layer fascia patchwork closure for the extended endoscopic transsphenoidal transtuberculum transplanum approach: Deep suturing technique and preliminary results. World Neurosurg..

[7] Hara T (2015). Cranial base repair using suturing technique combined with a mucosal flap for cerebrospinal fluid leakage during endoscopic endonasal surgery. World Neurosurg..

[8] Hadad G (2006). A novel reconstructive technique after endoscopic expanded endonasal approaches: Vascular pedicle nasoseptal flap. Laryngoscope.

[9] Kassam AB (2008). Endoscopic reconstruction of the cranial base using a pedicled nasoseptal flap. Neurosurgery.

[10] Horiguchi K (2010). Endoscopic endonasal skull base reconstruction using a nasal septal flap: Surgical results and comparison with previous reconstructions. Neurosurg. Rev..

[11] McCoul ED (2014). Long-term effectiveness of a reconstructive protocol using the nasoseptal flap after endoscopic skull base surgery. World Neurosurg..

[12] Teramoto S, Tahara S, Hattori Y, Morita A (2020). Skull base dural closure using a modified nonpenetrating clip device via an endoscopic endonasal approach: Technical note. Neurol. Med. Chir. (Tokyo).

[13] Wong J, Bhattacharya G, Vance SJ, Bistolarides P, Merchant AM (2013). Construction and validation of a low-cost laparoscopic simulator for surgical education. J. Surg. Educ..

[14] Caban AM (2013). Use of collapsible box trainer as a module for resident education. Jsls.

[15] Tokuyasu T (2018). Training system for endoscopic surgery aiming to provide the sensation of forceps operation. J. Robot. Mechatron..

[16] Xie T, Zhang X, Gu Y, Sun C, Liu T (2018). A low cost and stepwise training model for skull base repair using a suturing and knotting technique during endoscopic endonasal surgery. Eur. Arch. Otorhinolaryngol..

[17] Okuda T (2014). The chicken egg and skull model of endoscopic endonasal transsphenoidal surgery improves trainee drilling skills. Acta Neurochir (Wien).

[18] Kiyofuji S (2021). Development of integrated 3-dimensional computer graphics human head model. Oper. Neurosurg. (Hagerstown).

[19] Heredia-Pérez SA (2019). Virtual reality simulation of robotic transsphenoidal brain tumor resection: Evaluating dynamic motion scaling in a master-slave system. Int. J. Med. Robot..

[20] Shen Z (2020). The manufacturing procedure of 3D printed models for endoscopic endonasal transsphenoidal pituitary surgery. Technol. Health Care.

[21] Wen G (2016). A practical 3D printed simulator for endoscopic endonasal transsphenoidal surgery to improve basic operational skills. Childs Nerv. Syst..

[22] Ishii Y, Tahara S, Oyama K, Kitamura T, Teramoto A (2011). Easy slip-knot: A new simple tying technique for deep sutures. Acta Neurochir. (Wien).

[23] Marinho MM, Harada K, Morita A, Mitsuishi M (2020). SmartArm: Integration and validation of a versatile surgical robotic system for constrained workspaces. Int. J. Med. Robot.

[24] Morita A (2016). Medical engineering and microneurosurgery: Application and future. Neurol. Med. Chir. (Tokyo).

